# Comparison of Throat Washings, Nasopharyngeal Swabs and Oropharyngeal Swabs for Detection of SARS-CoV-2

**DOI:** 10.3390/v13040653

**Published:** 2021-04-10

**Authors:** Florian Hitzenbichler, Stilla Bauernfeind, Bernd Salzberger, Barbara Schmidt, Jürgen J. Wenzel

**Affiliations:** 1 Department of Infection Prevention and Infectious Diseases, University Medical Center Regensburg, 93053 Regensburg, Germany; Florian.Hitzenbichler@ukr.de (F.H.); Stilla.Bauernfeind@ukr.de (S.B.); Bernd.Salzberger@ukr.de (B.S.); 2 Institute of Medical Microbiology and Hygiene, University of Regensburg, 93053 Regensburg, Germany; Barbara.Schmidt@ukr.de; 3 Institute of Clinical Microbiology and Hygiene, University Medical Center Regensburg, 93053 Regensburg, Germany

**Keywords:** SARS-CoV-2, COVID-19, throat washing, nasopharyngeal swab, oropharyngeal swab, saliva, nucleic acid test, PCR, RT-qPCR, diagnostic sensitivity

## Abstract

Severe Acute Respiratory Syndrome Coronavirus 2 (SARS-CoV-2) RNA is detected by reverse-transcription quantitative real-time PCR (RT-qPCR) from respiratory specimens. This study compares throat washings (TW), nasopharyngeal swabs (NS) and oropharyngeal swabs (OS). A total of 102 samples from 34 adult patients with confirmed SARS-CoV-2 infection were analysed by RT-qPCR with absolute quantification. The median concentrations and diagnostic sensitivities were $5.8\times 10^{4}$ copies/mL, 85% (NS), $1.4\times 10^{4}$, 79% (OS) and $4.3\times 10^{3}$, 85% (TW). Concentration differences were significant between NS and TW (*P* = 0.019). Saliva (SA) was available from 21 patients (median $3.4\times 10^{3}$). OS and TW can be considered for SARS-CoV-2 diagnostics, although with slightly lower concentrations.

## 1. Introduction

Following the first appearance of a novel coronavirus (subsequently named SARS-CoV-2) at the end of December 2019 in Wuhan, China, a rapid worldwide spread occurred. The clinical presentation of infected individuals varies considerably, ranging from asymptomatic carriers to fatal multi-organ failure. Fast and reliable laboratory analysis from respiratory specimens is crucial for the placement and management of patients. The Robert Koch Institute in Germany recommends nasopharyngeal swabs for diagnosis of SARS-CoV-2 infection. The Centers for Disease Control and Prevention (CDC, Atlanta, USA) recommend nasopharyngeal or oropharyngeal swabs. Throat washing (gargling) is discussed as an alternative. Previous studies have reported various results on the diagnostic sensitivity of saliva or sputum [1,2,3,4]. Each sampling method offers advantages and disadvantages in clinical routine. For example, nasopharyngeal swabs are more difficult to obtain and currently affected by global supply shortages. On the other hand, throat washings are easy to perform, but could increase the risk of aerosolization or negatively influence the detection rate. Reliable data on the diagnostic performance of throat washing compared to nasopharyngeal or oropharyngeal swabs are scarce. We aimed to compare the diagnostic sensitivity and concentration of SARS-CoV-2-specific RNA as derived by three sampling methods: throat washings (TW), nasopharyngeal swabs (NS) and oropharyngeal swabs (OS). Moreover, saliva samples were analyzed from a subset of the study participants.

## 2. Materials and Methods

### 2.1. Subjects and Specimens

Between 27 April and 8 December 2020, thirty-four patients with coronavirus disease 2019 (COVID-19), who had been admitted to our non-intensive care medical ward, were included in this study. Inclusion criteria were: laboratory-confirmed SARS-CoV-2 infection (defined by a positive RT-qPCR result before inclusion) and age ≥ 18 years. All samples were collected by a single infectious diseases specialist in a defined sequence: (1) saliva (subset of 21 patients between May and December 2020), (2) oropharyngeal swab, (3) nasopharyngeal swab, and (4) throat washing. To obtain TW, patients were instructed to take 10 mL of medical grade saline or water in their mouth, gargle for 5–10 s (oscillating over the posterior pharyngeal wall) and transfer the liquid into a sterile test tube. NS and OS were obtained according to standard procedures. No combined swabs were collected. Only one nostril was probed for collecting NS.

### 2.2. Molecular Detection

All specimens were analyzed within 24 h after collection. Swab specimens were resuspended in 1 mL of medical grade saline before extraction. Nucleic acid was isolated from 200 µL of sample on an EZ1 Advanced XL workstation using the EZ1 Virus Mini Kit v2.0 (Qiagen, Hilden, Germany) with 100 µL elution buffer. A defined amount of MS2 bacteriophages was added during extraction and served as an internal control for isolation, reverse transcription and amplification. The concentration of SARS-CoV-2-specific RNA was determined by RT-qPCR. Briefly, 5 µL eluate was used for one-step reverse transcription and amplification in a total reaction volume of 30 µL using the TaqPath^TM^ 1-Step RT-qPCR Master Mix, CG (Thermo Fisher Scientific, Waltham, MA, USA). The PCR primers and fluorescent hydrolysis probe targeted the E-region of the viral genome [5]. Thermal cycling and real-time detection were performed on a StepOnePlus Real-Time PCR System (Thermo Fisher). The protocol was extended to allow absolute quantification of SARS-CoV-2 RNA as expressed in genome copies/mL (cp/mL). Therefore, standard curves were obtained by reverse transcription and amplification of a series of defined amounts of pre-characterized, in-vitro-transcribed and assay-specific RNA standards, generated as previously described for a different detection assay [6]. The specific target concentrations in the specimens were obtained by comparing the respective quantification cycles (Ct-values) to the standard curves. The analytical sensitivity (LoD_95_) of the assay was ≤300 cp/mL of specimen (corresponding to ≤3 copies per reaction).

### 2.3. Statistical Analysis

SARS-CoV-2 RNA levels in sample groups were compared. Undetectable viral loads were set at 100 RNA cp/mL and a decadic logarithmic ($log_{10}$) transformation of measurements was performed prior to the statistical analysis (example: $log_{10}\left(1.0\times 10^{5}\right)=5.0$). The Shapiro–Wilk test was used to test for normal distribution. Levene’s test was used to assess if samples were from populations with equal variances. The $log_{10}$-transformed concentrations of groups were first analyzed by a linear mixed-effects model (LME). The null hypothesis ($H_{0}$) assumed no difference in mean concentrations between groups. If the LME analysis yielded a non-significant result, $H_{0}$ could not be falsified and no further analysis followed. When LME yielded a significant result, at least one of the means of the groups was assumed to be different from the others ($H_{0}$ falsified). In this case, post-hoc comparisons based on the LME followed between each group. All statistical analyses were performed using *R*, version 4.0.4 (The *R* Foundation for Statistical Computing, Vienna, Austria). The *lmer()* function implemented in the *R*-package *lme4* was used for calculating the linear mixed-effects model. The *emmeans()* function from the *R*-package *lmerTest* was used for the post-hoc comparisons. *P* values of < 0.05 were considered to be statistically significant.

## 3. Results

Thirty-four individuals aged 22–83 years (mean 57.5 years) participated in the study. Eleven (32%) were female. Most patients were admitted with symptoms consistent with COVID-19. Three patients had no COVID-19 symptoms, were admitted for other medical issues and had a positive SARS-CoV-2 screening result. Only one patient (3%) was transferred to an intensive care unit and all patients survived. Detailed clinical and demographic characteristics are shown in Table 1. Diagnostic samples were collected 6.1 ± 3.7 days (±SD) after symptom onset (range 0–15 days). A total of 102 TW, NS and OS specimens were analyzed. Saliva (SA) was tested in a subset of 21 patients between May and December 2020.

**Figure 1:**
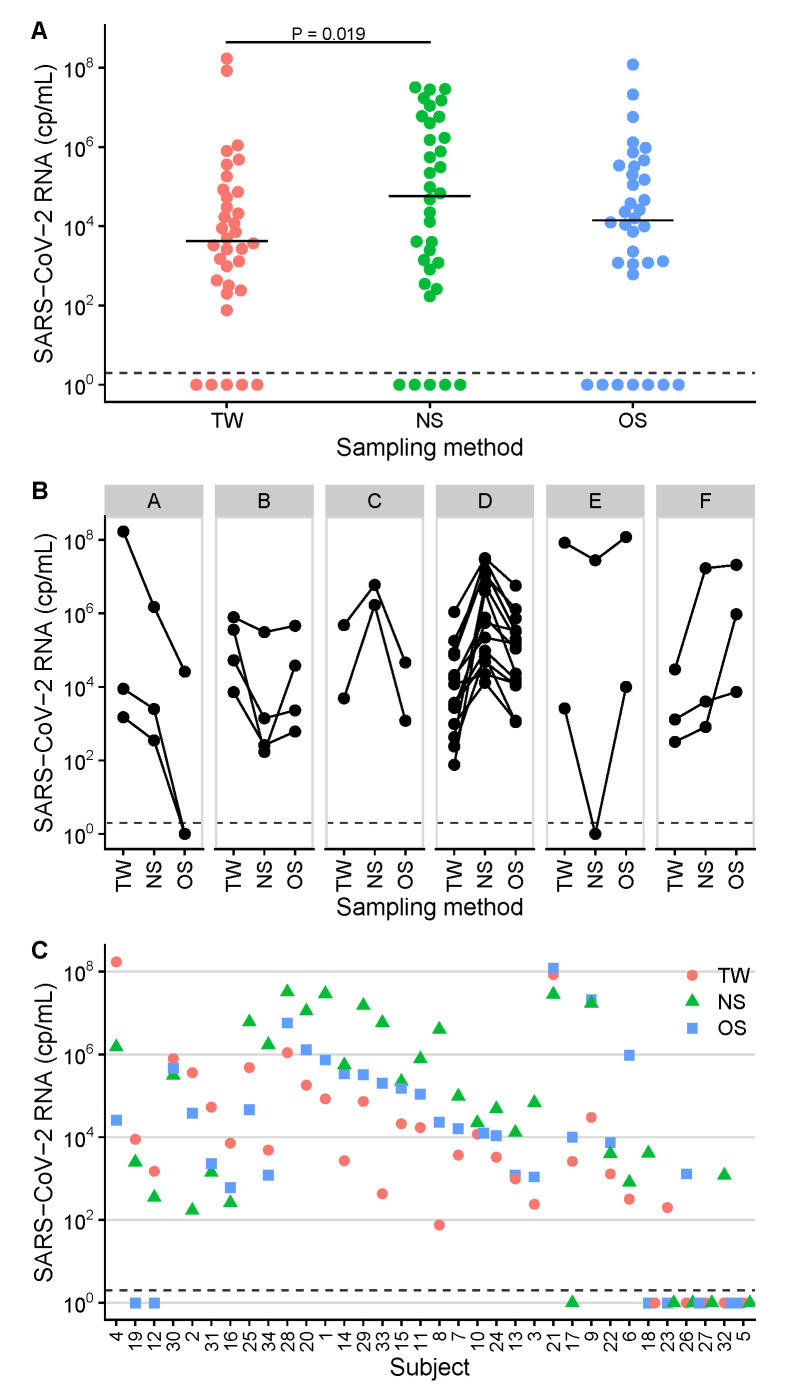
SARS-CoV-2 RNA concentrations in samples of 34 study participants. Measurements are separated into sampling site groups (TW, throat washing; NS, nasopharyngeal swab; OS, oropharyngeal swab). Samples with the result *not detected* were set to 10^0^ cp/mL and are plotted below the dashed lines. (**A**) Levels of SARS-CoV-2 viral loads across sampling sites. The respective median concentrations are shown as horizontal lines. The statistically significant difference between the groups was assessed by post-hoc tests based on the linear mixed-effects model and is indicated by a horizontal line with the corresponding *P* value < 0.05 on top of the data panels. (**B**) Concentration patterns *A–F* as found by combinatorial analysis of the quantitative results. Data from six subjects with ≤1 positive result are not shown. (**C**) comparative SARS-CoV-2 RNA quantification plots, sorted by concentration pattern and decreasing median viral load. Subject identification numbers are indicated on the x-axis.

The diagnostic sensitivity was assessed with regard to a positive RT-qPCR reference result before inclusion in the study. The median time between this pre-test and inclusion was 1.5 days (IQR 1–3, range 0–10). Overall, the diagnostic sensitivity was 85% for NS (29/34 positive), 79% for OS (27/34 positive) and 85% for TW (29/34 positive). SARS-CoV-2 RNA ranged from 0 (not detected) to $1.7\times 10^{8}$ cp/mL (Table 2, Figure 1).

The median concentrations were $5.8\times 10^{4}$ for NS, $1.4\times 10^{4}$ for OS and $4.3\times 10^{3}$ cp/mL for TW, respectively (Table 2, Figure 1, panel A). The $log_{10}$-transformed SARS-CoV-2 RNA levels were compared between the three sample groups NS, OS and TW by a linear mixed-effects model. The result indicated at least one significant difference in mean concentrations between the groups (*P* < 0.05). The following post-hoc comparisons showed a significant difference between NS and TW (*P* = 0.019, Figure 1, panel A). The combinations NS–OS (*P* = 0.073) and OS–TW (*P* = 0.56) did not differ significantly. Six different concentration patterns (designated *A* to *F*) were found by combinatorial analysis of the quantitative results (Figure 1, panel B; six patients with ≤1 positive result are not shown). The most prevalent pattern was NS > OS > TW (14/28; 50%; pattern *D*), followed by TW > OS > NS (4/28; 14%, pattern *B*). NS yielded the highest concentration in 18 (56%), TW in 8 (25%) and OS in 6 (19%) of 32 patients (Table 2, Figure 1, panel C).

**Figure 2:**
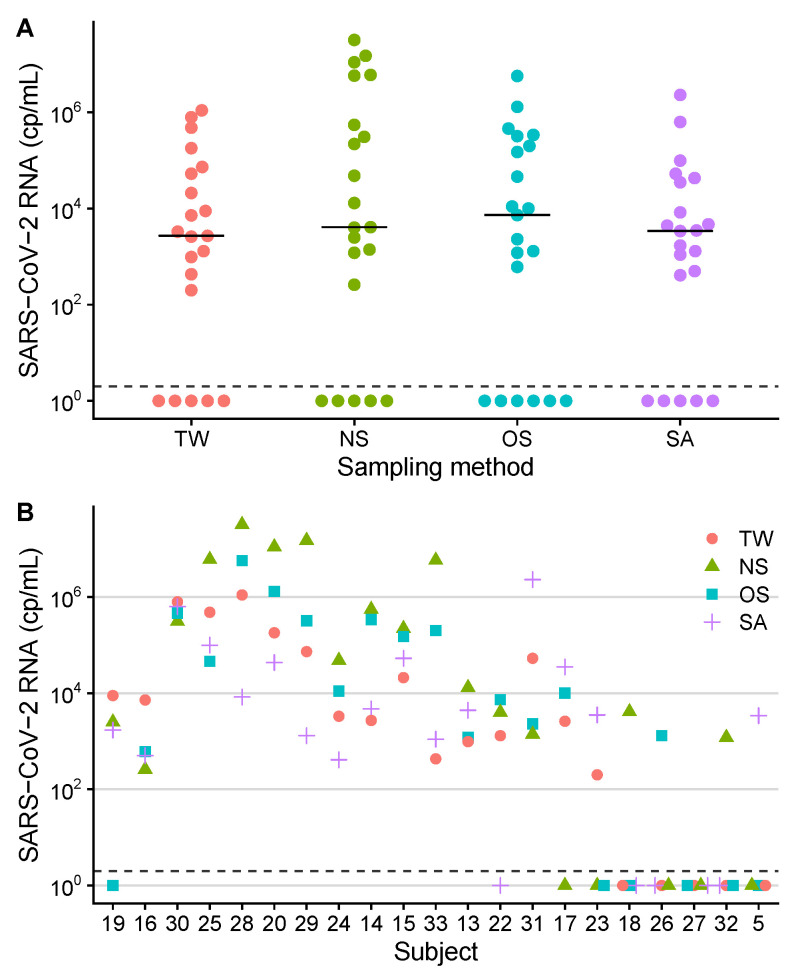
SARS-CoV-2 RNA concentrations in samples of 21 study participants. Measurements are separated into sampling site groups (TW, throat washing; NS, nasopharyngeal swab; OS, oropharyngeal swab; SA, saliva). Connected datapoints represent results from one patient. Samples with the result *not detected* were set to 10^0^ cp/mL and are plotted below the dashed lines. (**A**) Levels of SARS-CoV-2 viral loads across sampling sites. The respective median concentrations are shown as horizontal lines. No statistically significant differences between the four sampling groups were found by linear mixed-effects model analysis. (**B**) comparative SARS-CoV-2 RNA quantification plots, sorted by concentration pattern and decreasing median viral load. Subject identification numbers are indicated on the x-axis.

The subgroup of 21 patients, where all sample materials (including SA) were available, was analyzed separately. The diagnostic sensitivities were 76% for NS, TW and SA (16/21 positive, respectively) and 71% for OS (15/21 positive). The SARS-CoV-2 RNA load in saliva ranged from 0 (not detected) to $2.3\times 10^{6}$ cp/mL (Table 2, Figure 2).

The respective median concentrations in this subset were $7.3\times 10^{3}$ cp/mL for OS, $4.1\times 10^{3}$ for NS, $3.4\times 10^{3}$ for SA, and $2.7\times 10^{3}$ for TW. $Log_{10}$-transformed SARS-CoV-2 RNA levels were compared between the four sample groups: NS, OS, TW and SA. No statistically significant difference was found (*P* = 0.31, LME model) and no post-hoc comparisons were performed. In the combinatorial analysis, the most prevalent concentration patterns were NS > OS > TW > SA (4/15; 27%) and NS > OS > SA > TW (3/15; 20%).

## 4. Discussion

In this study, we *(a)* aimed to compare levels of SARS-CoV-2-specific RNA derived by three sampling methods: throat washings (TW), nasopharyngeal swabs (NS) and oropharyngeal swabs (OS). Our results showed significantly higher concentrations in NS as compared to TW, but NS–OS and OS–TW did not differ significantly. The highest diagnostic sensitivities were found for NS and TW (85%), followed by OS (79%). Moreover, we *(b)* investigated saliva (SA) in a subgroup analysis: TW, NS, OS and SA did not differ significantly.

Our first finding of a significant difference between NS and TW is supported by RT-qPCR based quantitative analysis of SARS-CoV-2 RNA levels in 102 specimens from 34 hospitalized patients with COVID-19 and moderate disease intensity. Our second finding is based on data from a subgroup covering 84 specimens from 21 patients.

The current standard for molecular COVID-19 diagnostics involves testing NS specimens by RT-qPCR. Other specimens have been investigated in previous studies. Guo et al. found TW to be significantly superior to NS in paired samples [8]. These authors proposed TW as a promising candidate for SARS-CoV-2 screening and monitoring due to its noninvasiveness and reliability. However, the limitations of this study were the small sample size (7 patients) and a sampling time long after symptom onset (median 53 days, range 48–57 days), which might have contributed to the low positive detection rate. The results of our study do not confirm that TW is superior to NS. We found the median concentration of NS being 13–times ($1.1\;log_{10}$) higher than in TW. This difference could—at least partially—be due to the higher volume (10 mL) of TW samples and the resulting dilution. In line with this observation, our data also suggest the use of NS samples as preferential material for antigen testing, which requires higher viral loads for detection.

Wyllie et al. investigated saliva as an alternative to standard NS specimens [2]. These authors detected more SARS-CoV-2 RNA copies in the saliva than in the NS specimens (70 patients; mean $3.8\times 10^{5}$ vs. $8.5\times 10^{4}$ cp/mL). They conclude that saliva and NS have at least similar sensitivity in the detection of SARS-CoV-2 during the course of hospitalization. The results of our study do not confirm this observation. In contrast, we found virtually the same median viral load from NS samples compared to SA ($4.1\times 10^{3}$ vs. $3.4\times 10^{3}$ cp/mL; 1.2–times higher = $0.1\;log_{10}$) in a subset of 21 patients. Variations in nasopharyngeal sampling may be an explanation for this discrepancy: The study by Wyllie et al. included NS collected by many health professionals while in our study all samples were collected by a single trained infectious disease specialist. In this context, a recently published study by Jamal et al. [3] reported a lower diagnostic sensitivity of SA (72%) as compared to NS (89%).

It is an ongoing matter of debate, if collecting combined OS–NS or OS–nares samples [9,10] can improve the positive detection rate. Our study did not address this question directly, but the quantitative comparison of NS and OS per patient revealed a higher concentration of OS specimens in 10 of 34 (29%) patients. It could be speculated whether collecting a combined specimen has the potential to improve the diagnostic sensitivity as compared to NS alone.

There are some limitations to our study. First, we investigated adult COVID-19 patients with moderate disease from a non-intensive care medical ward. Therefore, our results may not be fully transferable to other groups, like children, outpatients or critically ill patients. However, we assume a comparable situation in adult outpatients with early disease stages, because the mean time since symptom-onset was only ∼6 days in our study. Second, only one nostril was probed for collecting NS, which might have negatively influenced the analytical sensitivity. Third, the collection of specimens took place between April and December 2020 in Southern Germany. The spectrum of circulating SARS-CoV-2 strains likely has changed since that time and we cannot exclude potential effects on the results.

## 5. Conclusions

In conclusion, our study found the highest SARS-CoV-2 RNA concentrations in NS and comparable diagnostic sensitivities in all sampling groups. Our demonstration of only moderately lower viral loads in OS and TW indicates that they can be considered as alternatives for SARS-CoV-2 diagnostics instead of NS—which are more difficult to obtain, often not tolerated well by patients and affected by global supply shortages. Furthermore, TW could allow patient self-sampling, thereby reducing the risk of infection for medical personnel.

## Figures and Tables

**Table 1:** Demographic and Clinical Details of the Study Subjects.

Patient	Age, y	Sex	Oxygen Therapy	ICU Therapy	Days Hospi- talized	Charlson Score ^a^	CT- Infiltrates ^b^	Days Sympto- matic
1	77	M	+	−	18	6	+	4
2	55	F	+	−	22	4	+	10
3	56	M	−	−	6	10	−	1
4	58	F	−	−	25	4	+	1
5	60	F	+	−	19	2	+	11
6	22	M	+	−	4	0	+	8
7	67	M	−	−	30	3	−	0
8	39	F	−	−	2	2	−	6
9	61	M	+	+	34	8	+	10
10	46	M	−	−	6	0	+	8
11	48	M	−	−	1	1	+	3
12	38	M	+	−	9	0	+	7
13	39	F	−	−	4	0	+	4
14	34	M	−	−	4	0	NA	4
15	57	M	+	−	6	2	−	6
16	58	M	−	−	7	1	+	11
17	65	M	−	−	7	5	+	1
18	67	M	−	−	13	4	+	3
19	48	F	−	−	8	7	+	7
20	46	M	−	−	2	0	NA	8
21	78	F	+	−	28	11	+	7
22	83	M	−	−	14	7	NA	2
23	49	M	+	−	13	2	+	15
24	74	F	+	−	4	2	+	9
25	77	M	+	−	31	7	+	0
26	45	F	+	−	7	1	+	7
27	59	M	+	−	7	2	+	9
28	73	F	−	−	11	3	NA	2
29	68	F	+	−	19	3	+	5
30	72	M	+	−	5	3	+	5
31	43	M	+	−	6	0	+	9
32	71	M	+	−	5	4	+	12
33	76	M	+	−	13	6	+	4
34	45	M	+	−	10	2	+	7
Summary ^c^	Mean 57.5	F 32%	56%	3%	Median 7.5	Median 2.5	87%	Mean 6.1
	SD 14.9	M 68%			IQR 5.25–17	IQR 1–4.75		SD 3.7

^a^ Calculated as described by Charlson et al. [7]. ^b^ Computed tomography scans of the thorax showing pulmonary infiltrates compatible with Coronavirus disease 2019. ^c^ Statistical data in the summary line are shown as percentages, mean and standard deviations (SD) or median and interquartile ranges (IQR). Abbreviations: +, present; −, absent; CT, computed tomography; F, female; ICU, intensive care unit; M, male; NA, not available; y, years.

**Table 2:** Quantitative Results (SARS-CoV-2 RNA copies per milliliter).

Patient	TW	NS	OS	SA
1	$8.4\times 10^{4}$	$2.9\times 10^{7}$	$7.3\times 10^{5}$	NA
2	$3.6\times 10^{5}$	$1.7\times 10^{2}$	$3.8\times 10^{4}$	NA
3	$2.4\times 10^{2}$	$6.8\times 10^{4}$	$1.1\times 10^{3}$	NA
4	$1.7\times 10^{8}$	$1.5\times 10^{6}$	$2.6\times 10^{4}$	NA
5	0	0	0	$3.4\times 10^{3}$
6	$3.2\times 10^{2}$	$8.1\times 10^{2}$	$9.5\times 10^{5}$	NA
7	$3.7\times 10^{3}$	$9.7\times 10^{4}$	$1.6\times 10^{4}$	NA
8	$7.6\times 10^{1}$	$4.0\times 10^{6}$	$2.3\times 10^{4}$	NA
9	$3.0\times 10^{4}$	$1.7\times 10^{7}$	$2.1\times 10^{7}$	NA
10	$1.2\times 10^{4}$	$2.2\times 10^{4}$	$1.3\times 10^{4}$	NA
11	$1.7\times 10^{4}$	$7.7\times 10^{5}$	$1.1\times 10^{5}$	NA
12	$1.5\times 10^{3}$	$3.5\times 10^{2}$	0	NA
13	$9.8\times 10^{2}$	$1.3\times 10^{4}$	$1.2\times 10^{3}$	$4.4\times 10^{3}$
14	$2.7\times 10^{3}$	$5.5\times 10^{5}$	$3.4\times 10^{5}$	$4.7\times 10^{3}$
15	$2.1\times 10^{4}$	$2.2\times 10^{5}$	$1.5\times 10^{5}$	$5.3\times 10^{4}$
16	$7.2\times 10^{3}$	$2.6\times 10^{2}$	$6.1\times 10^{2}$	$5.0\times 10^{2}$
17	$2.6\times 10^{3}$	0	$1.0\times 10^{4}$	$3.5\times 10^{4}$
18	0	$4.1\times 10^{3}$	0	0
19	$8.9\times 10^{3}$	$2.5\times 10^{3}$	0	$1.7\times 10^{3}$
20	$1.8\times 10^{5}$	$1.1\times 10^{7}$	$1.3\times 10^{6}$	$4.3\times 10^{4}$
21	$8.4\times 10^{7}$	$2.8\times 10^{7}$	$1.2\times 10^{8}$	NA
22	$1.3\times 10^{3}$	$4.0\times 10^{3}$	$7.3\times 10^{3}$	0
23	$2.0\times 10^{2}$	0	0	$3.5\times 10^{3}$
24	$3.3\times 10^{3}$	$4.8\times 10^{4}$	$1.1\times 10^{4}$	$4.1\times 10^{2}$
25	$4.8\times 10^{5}$	$6.0\times 10^{6}$	$4.6\times 10^{4}$	$9.9\times 10^{4}$
26	0	0	$1.3\times 10^{3}$	0
27	0	0	0	0
28	$1.1\times 10^{6}$	$3.2\times 10^{7}$	$5.7\times 10^{6}$	$8.3\times 10^{3}$
29	$7.3\times 10^{4}$	$1.5\times 10^{7}$	$3.2\times 10^{5}$	$1.3\times 10^{3}$
30	$7.9\times 10^{5}$	$3.1\times 10^{5}$	$4.6\times 10^{5}$	$6.3\times 10^{5}$
31	$5.3\times 10^{4}$	$1.4\times 10^{3}$	$2.3\times 10^{3}$	$2.3\times 10^{6}$
32	0	$1.2\times 10^{3}$	0	0
33	$4.3\times 10^{2}$	$5.8\times 10^{6}$	$2.0\times 10^{5}$	$1.1\times 10^{3}$
34	$4.9\times 10^{3}$	$1.7\times 10^{6}$	$1.2\times 10^{3}$	NA
Sensitivity ^a^	85%	85%	79%	76%
Summary ^b^	Median $4.3\times 10^{3}$	Median $5.8\times 10^{4}$	Median $1.4\times 10^{4}$	Median $3.4\times 10^{3}$
	IQR $3.5\times 10^{2}$ – $6.8\times 10^{4}$	IQR $9.1\times 10^{2}$ – $3.4\times 10^{6}$	IQR $1.1\times 10^{3}$ – $2.9\times 10^{5}$	IQR $4.1\times 10^{2}$ – $3.5\times 10^{4}$

^a^ Diagnostic sensitivity calculated as proportion of subjects with a positive test result among the total number of subjects analyzed. ^b^ Statistical data in the summary line are shown as median and interquartile ranges (IQR). Abbreviations: NA, not available; NS, nasopharyngeal swab; OS, oropharyngeal swab; SA, saliva; SARS-CoV-2, severe acute respiratory syndrome coronavirus 2; TW, throat wash.

## Data Availability

The data generated for this publication have been included in the current manuscript.

## Abbreviations

The following abbreviations are used in this manuscript:

CDC	Centers for disease control and prevention, Atlanta, GA, USA
COVID-19	Coronavirus disease 2019
Cp/mL	Copies per milliliter
CT	Computed tomography
F	Female
$H_{0}$	Null hypothesis
ICU	Intensive care unit
IQR	Interquartile range
LME	Linear mixed-effects model
LoD_95_	Limit of detection (95%)
M	Male
NA	Not available
NS	Nasopharyngeal swabs
OS	Oropharyngeal swabs
PCR	Polymerase chain reaction
RT-qPCR	Reverse-transcription quantitative real-time PCR
SARS-CoV-2	Severe acute respiratory syndrome coronavirus 2
SD	Standard deviation
TW	Throat washings
Y	Years
